# Brazilian Group of Gastrointestinal Tumours’ consensus guidelines for the management of oesophageal cancer

**DOI:** 10.3332/ecancer.2021.1195

**Published:** 2021-03-02

**Authors:** Duilio R Rocha-Filho, Renata D’Alpino Peixoto, Rui F Weschenfelder, Juliana F M Rego, Rachel Riechelmann, Anelisa K Coutinho, Gustavo S Fernandes, Alexandre A Jacome, Aline C Andrade, Andre M Murad, Celso A L Mello, Diego S C G Miguel, Diogo B D Gomes, Douglas J Racy, Eduardo D Moraes, Eduardo H Akaishi, Elisangela S Carvalho, Evandro S Mello, Fauze Maluf Filho, Felipe J F Coimbra, Fernanda C Capareli, Fernando F Arruda, Fernando M A C Vieira, Flavio R Takeda, Guilherme C C Cotti, Guilherme L S Pereira, Gustavo A Paulo, Héber S C Ribeiro, Laercio G Lourenco, Marcela Crosara, Marcelo G Toneto, Marcos B Oliveira, Maria de Lourdes Oliveira, Maria Dirlei Begnami, Nora M Forones, Osmar Yagi, Patricia Ashton-Prolla, Patricia B Aguillar, Paulo C G Amaral, Paulo M Hoff, Raphael L C Araujo, Raphael P Di Paula Filho, Rene C Gansl, Roberto A Gil, Tulio E F Pfiffer, Tulio Souza, Ulysses Ribeiro, Victor Hugo F Jesus, Wilson L Costa, Gabriel Prolla

**Affiliations:** 1 Hospital Universitário Walter Cantídio, 60430-372 Fortaleza, Brazil; 2 Grupo Oncologia D’Or, 04535-110 São Paulo, Brazil; 3 Grupo Oncoclínicas, 04543-906 São Paulo, Brazil; 4 Hospital Moinhos de Vento, 90035-000 Porto Alegre, Brazil; 5 Hospital Universitário Onofre Lopes, 59012-300 Natal, Brazil; 6 AC Camargo Cancer Center, 01525-001 Sao Paulo, Brazil; 7 Clínica AMO, 41.950-640 Salvador, Brazil; 8 Hospital Sírio Libanês, 01308-050 São Paulo, Brazil; 9 Laboratório Personal, 30130-090 Fortaleza, Brazil; 10 Hospital Israelita Albert Einstein, 05652-900, São Paulo, Brazil; 11 Hospital Beneficência Portuguesa de São Paulo, 01323-001 São Paulo, Brazil; 12 Faculdade de Medicina da Universidade de São Paulo, 01246903 São Paulo, Brazil; 13 Hospital São Rafael, 41253-190 Salvador, Brazil; 14 Américas Oncologia, 22775-001 Rio de Janeiro, Brazil; 15 Centro de Oncologia do Paraná, 81200-100 Curitiba, Brazil; 16 Universidade Federal de São Paulo, 04040-003 São Paulo, Brazil; 17 Hospital São Lucas da PUCRS, 90610-000 Porto Alegre, Brazil; 18 Faculdade de Ciências Médicas da Santa Casa de São Paulo, 01238-010 São Paulo, Brazil; 19 Hospital de Clínicas de Porto Alegre, 90035-903 Porto Alegre, Brazil; 20 Hospital Alemão Oswaldo Cruz, 01323-020 São Paulo, Brazil; 21 Hospital Aliança de Salvador, 41920-900 Salvador, Brazil

**Keywords:** oesophageal cancer, gastroesophageal cancer, guidelines

## Abstract

Oesophageal cancer is among the ten most common types of cancer worldwide. More than 80% of the cases and deaths related to the disease occur in developing countries. Local socio-economic, epidemiologic and healthcare particularities led us to create a Brazilian guideline for the management of oesophageal and oesophagogastric junction (OGJ) carcinomas. The Brazilian Group of Gastrointestinal Tumours invited 50 physicians with different backgrounds, including radiology, pathology, endoscopy, nuclear medicine, genetics, oncological surgery, radiotherapy and clinical oncology, to collaborate. This document was prepared based on an extensive review of topics related to heredity, diagnosis, staging, pathology, endoscopy, surgery, radiation, systemic therapy (including checkpoint inhibitors) and follow-up, which was followed by presentation, discussion and voting by the panel members. It provides updated evidence-based recommendations to guide clinical management of oesophageal and OGJ carcinomas in several scenarios and clinical settings.

## Introduction

Oesophageal cancer is among the ten most common types of cancer worldwide. More than 80% of the cases and deaths related to the disease occur in developing countries. It is usually about three to four times more common in males than in females, although high mortality rates in both genders are observed [1]. In Brazil, the National Cancer Institute estimates 11,390 new oesophageal cancer cases in each year of 2020–2022 triennium [2]. Oesophageal cancer is usually diagnosed in advanced stages, presenting locoregional invasion or distant metastasis, which makes the prognosis particularly poor in most cases.

## Objective

The Brazilian Group of Gastrointestinal Tumours (GTG) organised this report aiming to consolidate the opinion of Brazilian specialists on specific topics related to the diagnosis, staging and treatment of oesophageal cancer. This document was prepared based on an extensive review of each item, followed by the presentation, discussion and voting by the panel members. It provides updated evidence-based recommendations to guide clinical management of oesophageal and oesophagogastric junction OGJ carcinomas. However, GTG emphasises that the clinical judgment of the physician must prevail as the patient individuality is a critical aspect of medical examination.

## Panel composition

A panel of 50 physicians with different backgrounds, including radiology, pathology, endoscopy, nuclear medicine, genetics, oncological surgery, radiotherapy and clinical oncology, were invited to collaborate. All experts are involved in the practice of the diagnosis, management and treatment of patients with oesophageal cancer. Considering the regional diversity of Brazil, the GTG invited specialists from various regions, including representatives from Bahia, Ceara, Federal District, Minas Gerais, Rio Grande do Norte, Rio Grande do Sul, Rio de Janeiro and Sao Paulo. All members of the panel followed the recommendations of the Federal Council of Medicine concerning disclosure of potential conflicts of interest.

## The methodology used to develop the consensus

Topics were organised by the GTG and distributed among members a few months in advance of presentation and voting. All specialists did a comprehensive literature review, following the GTG’s standard guidelines on how each topic should be addressed. All panel members were instructed to describe the level of evidence and strength of recommendation, as shown in Table 1. During the face-to-face meeting, the participants presented a summary of each of the reviewed topics, followed by discussion and voting when no consensus was reached. In this report, the recommendations on different subjects may guide the clinical management in several scenarios and clinical settings. In specific topics, the recommendation may point out the lack of unanimity about the level of evidence or strength of recommendation.

## Disclaimer

All recommendations were constructed upon scientific evidence. Readers must contextualise each recommendation according to the technology availability in their clinical routine.

## Topics discussed

### Heredity

#### When to suspect hereditary syndromes in oesophageal cancer and OGJ and how to assess the patient?

There is no substantial evidence to support the investigation of hereditary syndromes in oesophageal cancer [3].

### Staging

**What tests are necessary for the diagnosis and staging of oesophageal cancer and OGJ?**

***Recommendations***

- Upper digestive endoscopy (UDE) with biopsy is the gold standard for the diagnosis of oesophageal cancer. EVIDENCE LEVEL I, RECOMMENDATION A.
- Computed tomography (CT) of the chest and abdomen with intravenous contrast should be carried out. It is unnecessary if positron emission tomography–CT (PET/CT) is already carried out. EVIDENCE LEVEL I, RECOMMENDATION A.
- CT of the pelvis should be carried out, if clinically indicated. EVIDENCE LEVEL I, RECOMMENDATION A.
- PET is a preferable test in the absence of M1 disease (PET-CT is preferred over PET). EVIDENCE LEVEL II, RECOMMENDATION B.
- In the absence of M1 disease, an echoendoscopy can be carried out with a fine needle biopsy when indicated. EVIDENCE LEVEL I, RECOMMENDATION A.
  - There is little evidence to support the indication of echoendoscopy after neoadjuvant therapy. EVIDENCE LEVEL II, RECOMMENDATION C.
- Laparoscopy is optional in patients with an OGJ tumour (in the absence of M1 disease). EVIDENCE LEVEL III, RECOMMENDATION B.
- Bone scintigraphy for T3N1 can be considered if PET is not available. EVIDENCE LEVEL III, RECOMMENDATION C.
- Bronchoscopy is indicated for tumours at or above the tracheal bifurcation. EVIDENCE LEVEL III, RECOMMENDATION B.
- The main objective of the initial patient evaluation is to determine whether the disease can be resected with curative intent.

UDE with biopsy is essential for the diagnosis and staging of the neoplasia. The Siewert tumour type should be assessed in all patients with adenocarcinomas involving the OGJ. Tumours whose epicentre is located within 1–5 cm above the anatomic OGJ are classified as Siewert type I. Siewert type II is defined as a carcinoma with the tumour epicentre located within 1 cm above and 2 cm below the OGJ. Tumours whose epicentre is located between 2 and 5 cm below the OGJ are defined as Siewert III and should be managed in accordance with the gastric cancer guidelines [4].

The next step is to carry out a multidetector CT of the chest and abdomen, which allows reconstruction in multiple planes [5]. If metastatic disease is not detected in CT scans, PET should be carried out. PET-CT detects up to 20% more distant metastasis in comparison to CT. PET-CT displays a superior diagnostic performance than PET in the metastasis screening. Also, PET-CT may lead to a change in the stage of disease in up to 40% of all cases. However, its pooled sensitivity for the detection of locoregional metastases was only 0.51 (95% confidence interval (CI) = 0.34–0.69) in a systematic review of 12 studies, since regional nodes are often obscured by the metabolic activity of primary tumour [6].

If metastatic disease or regional lymph node involvement is not diagnosed on CT or PET-CT, echoendoscopy to assess regional lymph nodes can be considered [7–11]. Echoendoscopy is the exam with the highest sensitivity and specificity for T and N staging of oesophageal and gastric cardia tumours. When an echoendoscopic assessment of the oesophagus is possible over the entire extent of the organ, the sensitivity of staging can reach 92% for the tumour and 88% for lymph node involvement [11].

However, echoendoscopy works better in the advanced rather than in the early stage of the disease. In a meta-analysis of 49 studies, pooled sensitivity to diagnose T1 and T4 tumours was 81.6% (95% CI = 77.8–84.9) and 92.4% (95% CI = 89.2–95.0), respectively [12]. Furthermore, the echoendoscopic discrimination between mucosal (T1a) and submucosal (T1b) lesions, which is important to select patients for endoscopic treatment, is only 80% accurate [13]. Endoscopic resection provides accurate information on the depth of tumour invasion and should be considered for early-stage tumours (cT1a and cT1b ≤2 cm) [13]. Some other conditions related to decreased accuracy of echoendoscopic staging are stenosis of the upper portion of the oesophagus (sensitivity 28% for tumours and 72% for lymph nodes assessment) [14] and previous neoadjuvant treatment (sensitivity 69% and specificity 52% for lymph nodes assessment). Therefore, whenever possible, carrying out echoendoscopy before neoadjuvant therapy is recommended [15].

We recommend bronchoscopy for tumours located at or above the carina in the initial staging, which can help in both surgery and radiotherapy treatments [16, 17]. A prospective study showed that even in tumours located below the tracheal bifurcation, bronchoscopy identified airway invasion in 6.5% of otherwise potentially operable patients [18].

In cases of malignant stenosis, we recommend dilation before echoendoscopy whenever possible to improve staging. This management may have an impact on up to 20% of the patients [19, 20]. Known tumour markers have no role in the diagnosis, staging or monitoring of patients with oesophageal and gastric cardia cancer.

### Pathology

**How should oesophageal tumours be histologically classified?**

***Recommendation***

- Histopathology studies of oesophageal tumours should follow the classification of the World Health Organization (WHO). EVIDENCE LEVEL I, RECOMMENDATION A.

Squamous cell carcinoma and adenocarcinoma correspond to approximately 95% of oesophageal cancer. Temporal trends in incidence vary for the two major histologic types of oesophageal cancer. Squamous cell carcinoma has become less common in several Western countries because of long-term reductions in tobacco use and alcohol consumption, and now accounts for less than 30% of all oesophageal cancers in the United States and Western Europe [2]. Although a rise in the incidence of adenocarcinoma has also been observed in Brazil, squamous cell carcinoma remains the most common histology in the country [2]. A cross-sectional study of 24,204 patients with oesophageal cancer registered between 2001 and 2010 in Brazilian hospital-based registries showed that 82% of the oesophageal cancer cases were squamous cell carcinomas, compared to only 9% of adenocarcinomas [21]. Among 565 patients with oesophageal carcinoma treated at a single centre in São Paulo between 2009 and 2011, 19% had the diagnosis of adenocarcinoma and 81% had squamous cell carcinomas [22].

Squamous cell carcinoma differs from the adenocarcinoma in several aspects, including epidemiology, tumour biology and clinical evolution. Currently, squamous cell carcinoma and adenocarcinomas are considered distinct entities.

According to the WHO’s report published in 2010 [23], oesophageal epithelial cancer is classified into the following:

- Squamous cell carcinoma;
- Verrucous carcinoma;
- Spindle cell carcinoma;
- Adenocarcinoma;
- Undifferentiated carcinoma;
- Mucoepidermoid carcinoma;
- Adenoid cystic carcinoma;
- Small cell carcinoma;
- Adenosquamous carcinoma;

When describing squamous cell, verrucous and spindle cell (squamous) carcinomas and adenocarcinoma subtypes, the tumour should be graded according to its degree of cell differentiation. Thus, the tumour can be classified as:

- Grade I: well-differentiated
- Grade II: moderately differentiated
- Grade III: poorly differentiated

Grade classification is not necessary for mucoepidermoid, adenoid cystic, small cell, adenosquamous and undifferentiated carcinomas, as the degree of differentiation is already implicit in each of these subtypes.

**Which information is critical in the histopathological study of oesophageal cancer?**

***Recommendations***

- Location and the disease extension. RECOMMENDATION A.
- Histological type. RECOMMENDATION A.
- Degree of differentiation. RECOMMENDATION A.
- Level of lymphatic, venous or perineural invasion. RECOMMENDATION A.
- The total number of dissected lymph nodes and the number of affected lymph nodes. RECOMMENDATION A.
- The margin status. RECOMMENDATION A.
- Staging according to tumour-node-metastasis (TNM) criteria. RECOMMENDATION A.

Concerning location and extension of the disease, the 8th edition of the AJCC staging manual^24^ divides oesophageal cancer into cervical, upper thoracic, middle thoracic, lower and abdominal thoracic and OGJ, defined as the tubular oesophagus attached to the stomach, and is measured from the top of the gastric folds. Tumours located in the cervical oesophagus are classified as cancer of the upper thoracic oesophagus. The abdominal oesophagus includes the lower thoracic oesophagus. Tumours involving the OGJ that have an epicentre within 2 cm proximal to the cardia or proximal to stomach should be classified as oesophageal cancer. Tumours whose epicentre is more than 2 cm from the OGJ (Siewert III) should be classified as gastric cancer, and therefore follow gastric TNM parameters for staging [24]. This reflects epidemiological findings that indicate that the prognosis of patients with oesophageal cancer and OGJ is correlated to T staging, histology, degree of differentiation and tumour location [25]. For data reporting purposes, Barrett’s oesophagus showing high-grade dysplasia in an oesophageal resection should be reported as *in situ* carcinoma.

The margins are segmented into proximal, distal and radial. The circumferential resection margin (CRM) represents the soft tissue closest to the deepest penetration of the tumour. The sections for evaluating the margins of the proximal and distal resections can be obtained in two orientations [24] as follows:

1. Facial sections parallel to the margin;
2. Longitudinal sections perpendicular to the margin.

The choice of the section impacts the analysis of the margin status. The distance from the tumour margin to the nearest resection margin should be measured if not compromised by invasive carcinoma. The proximal and distal resection margins should be assessed for Barrett’s oesophagus and squamous and glandular dysplasia if they are not compromised by invasive carcinoma [24].

Tumours resected by endoscopy are evaluated at the vertical margin. The lateral margins can be considered relevant in cases where the endoscopic dissection of submucosa was carried out in complete resection samples [24].

Adequate resection requires obtaining satisfactory margins, including the circumferential margin [26]. The definition of a positive CRM depends on the pathological reporting system used. The College of American Pathologists defines a positive CRM as the presence of tumour at the resection margin [27], whereas the United Kingdom Royal College of Pathologists defines a positive CRM as the presence of oesophageal cancer within 1 mm of the margin [28].

**How to grade the histological response to neoadjuvant treatment of oesophageal cancer and OGJ?**

***Recommendation***

- The therapeutic response must consider the percentage of viable neoplastic cells. RECOMMENDATION A.

The determination of the percentage of viable cells and the classification of non-viable cells, such as necrosis, fibrosis and mucus, is a reliable method to assess the tumour’s response [29]. Additionally, the 8th Edition of AJCC recommends the use of Ryan’s modified method, which has demonstrated good interobserver reproducibility and has prognostic value in rectal cancer [24] (Table 2). Also, the verification of lymph node remission corroborates the information of tumour response and provides relevant prognostic data, especially for those with a high response rate of the primary tumour [30].

**What is the minimum number of lymph nodes to be evaluated by the pathologist in the surgical specimen?**

***Recommendation***

- An assessment of at least 15 lymph nodes should be considered on the surgical specimen of oesophageal cancer. RECOMMENDATION A.

The extension of lymphadenectomy during the surgery of oesophageal cancer is of considerable debate. The minimum number of lymph nodes that should be resected has not been described in the literature. However, pieces of evidence associate better survival rates with more extended lymphadenectomies [31]. The enhancement of the accuracy in N staging requires the evaluation of 15 lymph nodes. It is important to note that the number of assessed lymph nodes impacts the accuracy of the pathology analysis. Therefore, resection of as many lymph nodes as possible is recommended.

**When and how should biomarkers be evaluated in gastric, oesophageal and OGJ cancer?**

***Recommendations***

- In biopsies of localised OGJ or oesophageal adenocarcinomas, the assessment of HER2 status is optional. EVIDENCE LEVEL V, RECOMMENDATION C.
- In biopsies of metastatic adenocarcinomas of the OGJ or oesophagus, with the intention of palliative treatment, HER2 status should be investigated. EVIDENCE LEVEL I, RECOMMENDATION A.
- The assessment of HER2 status in metastatic adenocarcinomas of the OGJ or oesophagus can be made in surgical specimens, biopsies or cell blocks of primary or metastatic tumours, by using immunohistochemistry and interpreted according to the recommended scoring system. Borderline cases (++) in immunohistochemistry must be confirmed by FISH test. EVIDENCE LEVEL I, RECOMMENDATION A.
- The evaluation of MSI should be carried out on samples of advanced oesophageal and OGJ carcinomas. EVIDENCE LEVEL III, RECOMMENDATION A.
- The assessment of MSI should be carried out on samples of early stage OGJ adenocarcinoma. EVIDENCE LEVEL IV, RECOMMENDATION B.
- Programmed cell death ligand 1 (PD-L1) assessment should be carried out on samples of advanced oesophageal and OGJ carcinomas. EVIDENCE LEVEL I, RECOMMENDATION A.
- Neurotrophic Tyrosine Receptor Kinase (NTRK) fusions testing should be carried out in advanced oesophageal and OGJ carcinomas. EVIDENCE LEVEL III, RECOMMENDATION A.

Overexpression of the growth factor receptor HER2 is reported in a subgroup of 7%–30% of patients with gastric and oesophageal adenocarcinoma. HER2 is a member of the HER family, which encompasses several growth factor receptors, including HER1, HER3 and HER4, all involved in the regulation of cell growth, proliferation and survival [32].

The rate of HER2 positivity in oesophageal adenocarcinoma varies considerably, but it may be slightly higher than in gastric carcinomas. In general, the overexpression of HER2 in oesophageal adenocarcinoma correlates with a worse prognosis. Different studies point to a correlation between HER2 overexpression and greater depth of tumour invasion, lymph node involvement, distant metastases and low survival rates [33].

The assessment of the status of HER2 is critical to define which patients with advanced disease could benefit from the use of trastuzumab. HER2 analysis can be carried out on surgical specimens, biopsies and cell blocks [34] (primary or metastatic tumour). Hofman *et al* [35] published a validated scoring system for assessing HER2 status in gastric cancer (Table 3).

The immediate fixation of approximately 20–30 minutes in buffered formalin is fundamental to histopathological analysis [36]. It is noteworthy that, in gastric and OGJ adenocarcinoma, the positivity of HER2 in the cell membrane found in immunohistochemistry tests is lateral and basal. No staining of the luminal surface of the cells can be observed. For diagnosis purposes in surgical specimens, it used to be necessary to find more than 10% of HER2 positive cells in the specimen. This criterion is, however, considered inadequate today, and the recommendation for diagnosis in surgical resection specimens should be the same for biopsy studies. A sample with a cluster of five positive cells is sufficient for diagnosis [35, 36].

During endoscopy, as many samples as possible of the suspected cancer lesion should be collected. It is recommended to obtain at least six samples to increase the accuracy, since false–negative results may occur if the amount of tissue is not adequate or minimal [36].

MSI is characterised by changes in regions with repeated DNA sequences (microsatellites), resulting from mutational inactivation or epigenetic silencing of DNA repair genes (for example, *MSH1*, *MSH2*, *MSH6* and *MLH1*). This is the primary cause of genetic instability observed in tumours. Neoplasms with MSI can stimulate the immune system, becoming susceptible to immunotherapy with the use of checkpoint inhibitors. Furthermore, OGJ adenocarcinomas with MSI seem less sensitive to perioperative chemotherapy [37]. However, there is no consensus about the best technique to test the presence of MSI. There is a high correlation between polymerase chain reaction and immunohistochemistry, and no significant difference has been found between them [38].

For oesophageal, OGJ and gastric tumours, PD-L1 expression should be determined by using the combined positive score (CPS) instead of tumour proportion score (TPS). CPS is the number of PD-L1 staining cells (tumour cells, lymphocytes and macrophages) divided by the total number of viable tumour cells and multiplied by 100. When compared to TPS, CPS is a more sensitive prognostic biomarker in these tumour types [39].

Although generally rare,* NTRK* gene fusions are oncogenic drivers of various adult and paediatric tumours, including oesophageal and OGJ carcinomas. Several methods have been used to detect *NTRK* gene fusions including immunohistochemistry, FISH, reverse transcriptase polymerase chain reaction (RT-PCR), and DNA- or RNA-based next-generation sequencing, as described elsewhere [40].

### Endoscopic resection

**When to consider endoscopic resection in oesophageal cancer or OGJ?**

***Recommendations***

- In patients with T1a tumours, oesophagectomy or endoscopic resection can be carried out to remove tumours locally. EVIDENCE LEVEL III, RECOMMENDATION A.
- Patients with a well-differentiated oesophageal or OGJ tumour limited to the epithelium or to the lamina propria should be treated with endoscopic resection. EVIDENCE LEVEL III, RECOMMENDATION A.
- In oesophageal tumours T1b, oesophagectomy is the primary treatment. EVIDENCE LEVEL I, RECOMMENDATION A.

Early oesophageal cancer is defined as high-grade dysplasia (previously called carcinoma *in situ*) or T1 carcinoma. T1 is divided into T1a (up to the mucosa muscle) and T1b (up to the submucosa) [41].

Cancer of the mucosa (T1a) includes high-grade dysplasia carcinoma *in situ* (T1am1) and tumours infiltrating the lamina propria (T1am2) or muscularis mucosa (T1am3). Submucosal carcinoma can be classified as T1bsm1, T1bsm2 or T1bsm3, depending on the invasion in the upper, middle or lower third of the submucosa in the surgical specimen [42, 43] or invasion of the superficial submucosa (less than 200 µm of the muscularis mucosa for squamous cell carcinoma and less than 500 µm for adenocarcinomas) [44].

The depth of the lesion in superficial oesophageal adenocarcinoma has a significant clinical value. In cases of superficial tumours limited to SM2, the probability of lymph node involvement is 11% and increases with the extension of the lesion in the mucosa [45]. Patients with SM3 tumours should be managed with oesophagectomy [46]. There is no consensus on the best therapeutic option in the treatment of early oesophageal tumours. Endoscopic resection certainly has fewer morbidity rates than oesophagectomy, although one may admit the possibility of recurrence. It is crucial that the pathological specimens are fixed adequately for pathology analysis.

### Non-metastatic disease: surgery for oesophageal cancer and OGJ

**What are the surgical options for the initial or early stages of oesophageal cancer and OGJ?**

***Recommendations***

- Patients with mucosa-limited oesophageal tumours (Tis or T1a) can be treated with endoscopic resection. EVIDENCE LEVEL III, RECOMMENDATION A.
- In patients with superficial oesophageal tumours (T1a), oesophagectomy or endoscopic resection can be carried out. EVIDENCE LEVEL III, RECOMMENDATION A.
- In T1bN0 or T2N0 oesophageal tumours, oesophagectomy is the first treatment option. EVIDENCE LEVEL I, RECOMMENDATION A.
- Oesophagectomy should be carried out in referral centres and by an experienced surgeon. EVIDENCE LEVEL IV, RECOMMENDATION A.
- Minimally invasive oesophagectomy has shown similar efficacy with less surgical complications and may be preferred over open approaches. EVIDENCE LEVEL I, RECOMMENDATION A.

Surgical mortality associated with oesophagectomy is lower in referral centres, with a large annual volume of major surgeries. The amount of surgeries per surgeon is also directly associated with mortality rates related to oesophagectomy [47, 48]. Thus, we recommend an experienced surgeon in a specialised reference centre to carry out an open or minimally invasive oesophagectomy. Different thresholds to define low- and high-volume institutions have been used, so that it is hard to establish a clear cut-off point [49]. Metzger *et al* [50] showed that a significant reduction of perioperative mortality can be achieved in centres that carry out more than 20 oesophagectomies per year.

Oesophagectomy has significantly higher morbidity and mortality than other highly complex surgeries. The 5-year survival rate for operated oesophageal cancer is about 20%, and the impact of surgical complications should always be considered. The prognostic factors with the most considerable influence on mortality and postoperative survival are R0 resection and lymph node staging [51]. The current data from squamous cell carcinoma suggest that a minimum proximal and distal margin of 3.5–5 cm is needed to decrease the risk of local recurrence. For adenocarcinoma of the oesophagus, the adequate margins are less clearly defined [52].

Minimally invasive surgery in early oesophageal cancer (via thoracoscopy) aims to reduce postoperative morbidity and mortality and may be a potentially advantageous alternative. It must follow the same oncological principles as open oesophagectomy, without compromising long-term survival and providing a better quality of life for the patient.

Several studies support the use of minimally invasive oesophagectomies. A randomised trial showed that patients undergoing minimally invasive oesophagectomy had a lower rate of pulmonary infections compared with patients treated with open oesophagectomy (12% versus 34%; relative risk 0.35; 95% CI = 0.16–0.78) [53]. No differences in disease-free or overall survival between arms were found. Another randomised trial compared robot-assisted minimally invasive thoracolaparoscopic oesophagectomy (RAMIE) with open oesophagectomy. RAMIE resulted in significant lower complications, better quality of life and better functional recovery, with similar oncological outcomes [54]. Accordingly, a French controlled trial found that hybrid minimally invasive oesophagectomy resulted in lower incidence of major complications than open oesophagectomy, without compromising overall and disease-free survival [55]. Furthermore, different meta-analyses support the safety and efficacy of minimally invasive surgery [56–59].

### Non-metastatic disease – chemotherapy and radiotherapy in oesophageal and OGJ cancer

**When and which neoadjuvant treatment should be indicated to oesophageal or OGJ cancer?**

***Recommendations***

Adenocarcinoma cT2 cN0:

- Perioperative chemotherapy is preferably the treatment of choice. EVIDENCE LEVEL I, RECOMMENDATION A.
- Upfront surgery is also an alternative. EVIDENCE LEVEL I, RECOMMENDATION B.
- Combined chemoradiotherapy with carboplatin and paclitaxel is another option. EVIDENCE LEVEL I, RECOMMENDATION B.

Squamous cell carcinoma cT2N0:

- Combining chemoradiotherapy with carboplatin and paclitaxel is preferably the treatment of choice. EVIDENCE I, RECOMMENDATION A.
- Upfront surgery is also an alternative. EVIDENCE I, RECOMMENDATION B.

Adenocarcinoma T3–T4a and/or N+:

- Either combined chemoradiotherapy with carboplatin and paclitaxel or perioperative chemotherapy are recommended. EVIDENCE I, RECOMMENDATION A.
- Preoperative chemoradiotherapy may be preferred in patients with Siewert I tumours, those with higher risk of positive margins or more susceptible to severe treatment-related adverse events. EVIDENCE V, RECOMMENDATION C
- Perioperative chemotherapy may be preferred in the management of Siewert III tumours, in patients with higher risk of distant relapse, such as those with multiple positive nodes, in patients who need rapid palliation of dysphagia or who have difficult access to radiation therapy. EVIDENCE V, RECOMMENDATION C
- FLOT is the preferred perioperative chemotherapy regimen for patients with good performance status. EVIDENCE I, RECOMMENDATION A.
- Perioperative chemotherapy with cisplatin and 5-fluorouracil (CF) is also a valid option, as well as oxaliplatin-based doublets FOLFOX or CAPOX. EVIDENCE I, RECOMMENDATION B.
- Induction chemotherapy followed by chemoradiotherapy may also be considered. EVIDENCE II, RECOMMENDATION C.

Squamous cell carcinoma T3–T4a and/or N+:

- Chemoradiotherapy with carboplatin and paclitaxel is recommended. EVIDENCE I, RECOMMENDATION A.

***Induction chemotherapy followed by surgery***

The theoretical advantages of this approach include better chances of tumour resection and reduced regional and distant micrometastases. Documented clinical response rates vary from 40% to 65%; the proportion of complete pathological response varies from 0% to 10%; and resectability rates range from 40% to 80% [60–65]. To date, there are four large prospective randomised, phase III studies comparing induction chemotherapy, followed by surgery versus isolated surgery in patients with locally advanced oesophageal cancer. In these studies, the 5-year survival rate varied between 23% and 38% in groups that were treated with chemotherapy, followed by surgery versus 17%–24% in groups of patients undergoing isolated surgery [66–70]. Two meta-analyses suggest a more significant benefit of induction chemotherapy in patients with squamous cell carcinoma versus adenocarcinoma [71, 72].

***Neoadjuvant chemoradiotherapy***

The poor long-term prognosis and the sensitising effect of cytotoxic agents on radiation led several researchers to evaluate chemoradiotherapy in the preoperative (neoadjuvant) setting. There are at least ten prospective, randomised, phase III studies that compare concomitant chemoradiotherapy, followed by surgery with isolated surgery in patients with potentially resectable oesophageal carcinoma [73–82]. Among these studies, the most important are the Dutch CROSS trial and CALGB 9781 trial [80, 83].

In the CROSS trial, Dutch researchers randomised 368 patients with potentially resectable oesophageal or OGJ carcinoma to receive preoperative chemoradiotherapy with paclitaxel 50 mg/m^2^ associated with carboplatin (area under the curve = 2) and concomitant radiation therapy (41.4 Gy for 5 weeks) or surgery alone. In this study, 75% of the patients had adenocarcinoma and 23% had squamous cell carcinoma. Besides, most patients had disease located in the third distal of the oesophagus or OGJ (82% of the patients). R0 resection rates were higher in patients undergoing neoadjuvant chemoradiotherapy (92% versus 69%), and 29% of those who received neoadjuvant treatment achieved a complete pathological response. After a median follow-up of 45.4 months, the median survival of patients receiving concomitant neoadjuvant chemoradiotherapy was statistically superior than in the group treated with surgery alone (49.4 months versus 24.0 months; hazard ratio (HR) = 0.657; *p* = 0.003) [83, 84]. An alternative regimen with 5-fluorouracil (5-FU) and cisplatin is optional in services where paclitaxel and carboplatin are not available [85].

CALGB 9781 was designed as a randomised trial of trimodal therapy versus surgery in 500 patients with oesophageal or OGJ cancer with stage I–III. Because of low recruitment, the study was closed prematurely, with only 56 patients included. Although the result is not statistically significant, 5-year survival rate was 39% versus 16% in favour of trimodal therapy [80].

There is only one phase III study that compares chemoradiotherapy with chemotherapy in the neoadjuvant setting in patients with OGJ adenocarcinoma. The German POET trial randomised 126 patients to 16 weeks of chemotherapy alone (cisplatin and 5-FU/leucovorin) versus 12 weeks of the same chemotherapy, followed by radiotherapy concomitant with cisplatin and etoposide. Both groups underwent subsequent surgical resection. The rate of complete pathological response was significantly superior in the group that received chemoradiotherapy (16% versus 2%). Likewise, a trend towards better survival in 3 years was observed in this group (47% versus 28%; *p* = 0.07). The POET findings support the neoadjuvant chemoradiotherapy strategy for patients with OGJ adenocarcinoma. However, it is essential to note that the study closed prematurely and, therefore, is underpowered to show consistent survival benefit [86].

Several meta-analyses that address the possible differences between neoadjuvant treatments and surgery alone in the two histological types of oesophageal carcinoma were published. In 2011, Kranzfelder *et al* [87] reported meta-analysis results that included 20 trials addressing neoadjuvant chemoradiotherapy or chemotherapy versus surgery alone. HR for overall survival was 0.81 (95% CI = 0.70–0.95; *p* = 0.008) after chemoradiotherapy and 0.93 (95% CI = 0.81–1.08; *p* = 0.368) after chemotherapy. Also, better R0 resection rates were observed with the two types of neoadjuvant therapy without increasing morbidity and mortality [87].

When investigating the same groups of patients, an updated meta-analysis that included 4,188 cases published by Sjoquist *et al* [88] showed reduced mortality with neoadjuvant chemoradiotherapy (HR = 0.78; 95% CI = 0.70–0.88; *p* < 0.0001), which means a survival benefit of 8.7% in 2 years, or a need to treat 11 patients to prevent one death. The benefit was similar in all histological subtypes (HR = 0.80 for squamous cell carcinoma; 95% CI = 0.68–0.93; *p* = 0.004 and HR = 0.75 for adenocarcinoma; 95% CI = 0.59–0.95; *p* = 0.02). The HR for the overall indirect comparison of all-cause mortality for neoadjuvant chemoradiotherapy versus neoadjuvant chemotherapy was 0.88 (95% CI = 0.76–1.01; *p* = 0.07) [88]. The benefit of concomitant chemoradiotherapy in oesophageal cancer is overwhelming. However, the need for surgery in patients who achieve a complete pathological response is still under debate.

A new approach to better select patients for chemoradiotherapy uses the PET/CT assessment. A phase II study evaluated early response with PET/CT after induction chemotherapy in 257 patients. Patients with oesophageal and OGJ adenocarcinoma with no response to the first neoadjuvant chemotherapy regimen received another chemotherapy regimen aiming to improve the complete pathological response. This strategy, however, still needs confirmation [34].

***Perioperative chemotherapy***

Oesophageal adenocarcinoma and squamous cell carcinoma behave as two distinct disease entities regarding epidemiological aspects, pathogenesis, natural history and prognosis.

Some authors consider that distal oesophageal, OGJ and gastric adenocarcinoma have the same biological response, and therefore share the treatment principles. Upon this reasoning, in 2006, the phase III MAGIC trial was published. The study randomly assigned 503 patients with resectable adenocarcinoma of the stomach, OGJ or lower oesophagus to three preoperative and three postoperative cycles of ECF regimen (epirubicin, cisplatin and 5-FU) or surgery alone. In 11% and 15% of the patients, the tumour site was in distal oesophagus and OGJ, respectively. Although only 42% of the patients completed the proposed treatment, an increase in both progression-free survival (HR = 0.66; 95% CI = 0.53–0.81; *p* < 0.001) and overall survival (HR = 0.75; 95% CI = 0.60–0.93; *p* = 0.009) was observed in favour of the group undergoing perioperative chemotherapy. The 25% reduction in the risk of death resulted in an increase in 5-year overall survival from 23% to 36%. In a subgroup analysis, the treatment benefited all patients, independently of the primary tumour site [67]. In line with these findings, the French study FNCLCC/FFCD, published in 2011, randomised 224 patients with adenocarcinoma of the distal oesophagus (*n* = 25), OGJ (*n* = 144) and stomach (*n* = 55) to receive perioperative chemotherapy with cisplatin and 5-FU regimen for two to three cycles, followed by surgery, and then three to four cycles of postoperative chemotherapy versus surgery alone. Only 50% of the patients in the experimental group received at least one postoperative chemotherapy cycle. Some authors observed a benefit in both progression-free survival in 5 years (34% versus 19%; HR = 0.65; *p* = 0.003) and overall survival in 5 years (38% versus 24%; HR = 0.69; *p* = 0.02) in favour of perioperative chemotherapy arm. Therefore, considering these two trials, perioperative chemotherapy is a solid option for patients with lower oesophageal adenocarcinoma and OGJ [70].

More recently, the German phase III FLOT4-AIO study randomised 716 patients to three preoperative cycles, followed by three postoperative cycles of ECF/ECX (epirubicin 50 mg/m^2^, cisplatin 60 mg/m^2^, on both D1 and 5-FU 200 mg/m^2^ as a continuous infusion or oral capecitabine 1,250 mg/m^2^ D1–21) every 3 weeks, or four preoperative cycles, followed by four postoperative cycles of FLOT (docetaxel 50 mg/m^2^, oxaliplatin 85 mg/m^2^, leucovorin 200 mg/m^2^ and 5-FU 2600 mg/m^2^ in a 24-hour infusion in D1) every 2 weeks. The primary endpoint was overall survival. Patients with gastric or OGJ adenocarcinoma (56% of the total patients) with stage ≥ cT2 and or N+ were eligible. FLOT regimen improved median overall survival from 35 months (ECF/ECX arm) to 50 months with a median follow-up of 43 months [89]. As shown in this study, FLOT supplanted the results of the previous MAGIC study and, therefore, when available and for fit patients, we recommend this scheme as the preferential neoadjuvant treatment modality.

*Post-hoc* analysis of MAGIC trial suggests that adjuvant or perioperative chemotherapy may not be helpful – and may even be deleterious – for oesophagogastric cancer patients with MSI. Although controversial, the MSI status may be used to select patients for perioperative treatment [37].

Some Asian studies have investigated the role of *preoperative* or perioperative chemotherapy in patients with squamous cell carcinoma. Adjuvant chemotherapy with cisplatin and fluorouracil was shown to increase disease-free survival over surgery alone, especially in the subgroup with lymph node metastasis, in the JCOG9204 randomised trial [90]. Subsequently, the JCOG9907 trial randomised patients with clinical stage II or III squamous cell carcinoma to undergo surgery, followed by two courses of cisplatin plus 5-FU, as in JCOG9204, or preceded by the same regimen. Preoperative treatment significantly increased 5-year overall survival (43% versus 55%) [91]. In Japan, preoperative chemotherapy is recommended for clinical stages II or III thoracic oesophageal cancer patients, based on data from the JCOG9907 study [92]. However, the role of preoperative or perioperative chemotherapy in squamous cell carcinoma is not defined in Western patients. Therefore, this panel does not recommend its use.

Both preoperative chemoradiotherapy and perioperative chemotherapy are reasonable options for the management of oesophageal adenocarcinoma T3–T4a and/or N+. Some patient and tumour characteristics may help defining the best treatment option. Chemoradiotherapy is commonly associated with higher cytotoxic activity than chemotherapy alone [86], although experimental arms in FLOT and CROSS trials have shown similar complete pathologic response rates in the adenocarcinoma population [83, 89]. In patients in need of cytoreduction and at risk of positive margins, including Siewert I tumours, the addition of radiotherapy is commonly considered. Acute toxicity data favour CROSS regimen over FLOT [83, 89], so that preoperative chemoradiation may be favoured in patients more susceptible to severe treatment-related adverse events . On the other hand, longer and more intense FLOT regimen is believed to be systemically more active than short-duration weekly carboplatin–paclitaxel used in the CROSS trial. Indeed, the impact of CROSS regimen in reducing distant metastasis seems to disappear over time [84]. In patients with higher risk of distant relapse, such as those with multiple nodes, perioperative FLOT may be preferred. FLOT may also be favoured in patients in need of rapid palliation of dysphagia or who have difficult access to radiotherapy centres.

**When should adjuvant immunotherapy be indicated to oesophageal or OGJ cancer?**

***Recommendation***

- Adjuvant nivolumab may be considered in patients with resected stage II/III oesophageal or OGJ cancer who received neoadjuvant chemoradiotherapy and have residual pathologic disease. EVIDENCE LEVEL II, RECOMMENDATION B.

CheckMate 577 is a phase III clinical trial that randomised patients who had pathologic residual disease after neoadjuvant chemoradiotherapy and R0 surgical resection to nivolumab or placebo for up to 1 year. Adjuvant nivolumab showed an improvement in median disease-free survival from 11.0 to 22.4 months (HR = 0.69; 96.4% CI = 0.56–0.86; *p* = 0.0003) [93]. Benefit was seen irrespective of histology or tumour PD-L1 expression, as assessed by Dako 28-8 pharmDx assay. Some caution is needed when interpreting these results, since overall survival data are not mature and median follow-up time is only 24 months. Updated data will be of great importance. Nevertheless, adjuvant nivolumab may be considered if available.

**When considered, what type of radiotherapy is recommended for patients with oesophageal or OGJ cancer?**

***Recommendations***

- In patients with oesophageal cancer (adenocarcinoma or epidermoid) stage T3–4a and/or N + M0, we recommend neoadjuvant chemoradiotherapy with carboplatin and paclitaxel, followed by surgery. EVIDENCE LEVEL I, RECOMMENDATION A.
- The total radiation dose is 41.4 Gy. EVIDENCE LEVEL I, RECOMMENDATION A.
- Definitive chemoradiotherapy is acceptable when surgery is not a good option considering *performance status* and comorbidity for patients with oesophageal adenocarcinoma with T3–4b and/or N+ M0 stages. EVIDENCE LEVEL II, RECOMMENDATION C.
- Definitive chemoradiotherapy is acceptable when surgery is not a good option considering *performance status* and comorbidity for patients with oesophageal squamous cell carcinoma with T3–4b and/or N+ M0 stages EVIDENCE LEVEL II, RECOMMENDATION B.
- Patients with T1b–4b N+ cervical oesophageal carcinoma may be treated with definitive chemoradiotherapy. EVIDENCE LEVEL IV, RECOMMENDATION B.
- Patients with adenocarcinoma stage T3–4a and/or N+ M0 may have surgery, followed by chemoradiotherapy. EVIDENCE LEVEL IV, RECOMMENDATION C.
- The chemotherapy regimen associated with radiotherapy for definitive chemoradiotherapy should preferably be composed of cisplatin and 5-FU. EVIDENCE LEVEL I, RECOMMENDATION B and paclitaxel and carboplatin EVIDENCE LEVEL II, RECOMMENDATION A.
- Chemotherapy associated with radiotherapy in stage T3–4b and/or N+ M0 oesophageal cancer may alternatively comprise oxaliplatin, irinotecan, docetaxel or capecitabine. EVIDENCE LEVEL III, RECOMMENDATION C.

**What is the best treatment option for locally advanced non-resectable, non-metastatic oesophageal or OGJ cancer?**

***Recommendations***

- Chemotherapy and radiotherapy can be indicated in selected patients with good *performance status* and in those who are inoperable or refuse surgery. EVIDENCE LEVEL II, RECOMMENDATION A.
- If the patient is not a candidate for definitive chemoradiotherapy, chemotherapy or palliative radiotherapy alone may be indicated. EVIDENCE LEVEL III, RECOMMENDATION B.
- The chemotherapy regimen associated with radiotherapy in unresectable locally advanced oesophageal cancer should preferably be carboplatin and paclitaxel. EVIDENCE LEVEL III, RECOMMENDATION A.
- Chemotherapy associated with radiation therapy in locally advanced oesophageal cancer may use, alternatively, platinum and fluoropyrimidine combination. EVIDENCE LEVEL I, RECOMMENDATION C.
- The total radiation dose is 50.4 Gy in the setting of definitive chemoradiotherapy non-resectable patients. EVIDENCE LEVEL I, RECOMMENDATION C.

The treatment of oesophageal cancer with locoregional invasion has evolved significantly in the past two decades. This topic addresses unresectable diseases for technical reasons due to a lack of clinical conditions or the patient’s refusal to surgery. The low complete response rates after local therapy alone led to the incorporation of systemic chemotherapy into the treatment, in an attempt to control distant micrometastases and to potentiate the local effects of radiation.

***Definitive chemoradiotherapy (without surgery)***

Cytotoxic agents and radiation therapy are combined to promote better outcomes in patients with advanced oesophageal cancer. The current use of platinum and 5-FU with radiotherapy appears to have significant radiation sensitising effect [94, 95]. Other agents, such as taxanes and irinotecan, have also been investigated for the same purposes [96, 97].

Two prospective, randomised, phase III trials, conducted by cooperative research groups, compared definitive radiotherapy with definitive concomitant chemoradiotherapy in patients with locally advanced oesophageal cancer [85, 98]. The first study (RTOG 85-01) compared chemotherapy with cisplatin (75 mg/m^2^ on day 1, weeks 1 and 5) and 5-FU (1,000 mg/m^2^ per day, days 1–4 of weeks 1 and 5) concomitant with radiotherapy (50 Gy in 2 Gy per day, over 5 weeks) versus conventional radiation therapy (64 Gy in 2 Gy per day, over 6.5 weeks) in patients with thoracic oesophageal cancer and with locoregional invasion. The group of patients randomised to chemoradiotherapy also received two additional cycles of chemotherapy after the end of radiotherapy. Surgery was not part of the treatment regimens. This trial closed prematurely with only 121 patients after an interim analysis that demonstrated a significant survival benefit in chemoradiotherapy arm. Median survival was 12.5 months for the combined group and 8.9 months for the group with isolated radiation therapy (*p* < 0.001) [85].

The superiority of definitive concomitant chemoradiotherapy over radiotherapy alone was confirmed by a second study, carried out by Eastern Cooperative Oncology Group (ECOG), which compared chemotherapy with 5-FU and mitomycin-C concomitant to radiotherapy (60 Gy) with radiation therapy alone (60 Gy) [98].

In 2010, a meta-analysis that included 19 randomised trials compared the definitive chemoradiotherapy approach (concomitant or in sequence) with radiotherapy alone as a treatment for oesophageal cancer with locoregional invasion. Chemotherapy concomitant with radiotherapy provided a significant reduction in mortality (HR = 0.73; 95% CI = 0.64–0.84). The results of trials with sequential chemoradiotherapy did not show significant benefit in survival or local disease control. The absolute gain in survival rates from combination therapy compared to radiotherapy alone was 9% in 1 year and 4% in 2 years [99].

Despite the absence of phase III studies with carboplatin and paclitaxel similar to the CROSS study in unresectable oesophageal cancer, the acceptable toxicity profile has made this scheme preferred in this clinical setting.

***Radiation therapy alone***

The radiotherapy treatment alone for oesophageal cancer is usually reserved for unresectable tumours in patients with a poor *performance status* to receive chemotherapy. In general, radiation monotherapy results in a median survival ranging from 6 to 12 months and a 5-year survival rate of less than 10% [100, 101]. Also, in phase III trials, the rates of local failure reach 84% [102, 103].

***Persistent or relapsed tumour after definitive chemoradiotherapy: salvage surgery***

**What is the role of salvage surgery?**

***Recommendation***

- Salvage surgery can be considered in selected patients with localised resectable oesophageal cancer after definitive chemoradiotherapy, if there is no distant recurrence. EVIDENCE LEVEL III, RECOMMENDATION B.

Two randomised trials did not identify a survival benefit of trimodal therapy over definitive chemoradiotherapy in squamous cell oesophageal cancer [104, 105]. Both neoadjuvant therapy with planned surgery and definitive chemoradiotherapy, followed by close observation can be recommended for those patients [106]. However, the non-operative strategy is associated with increased locoregional recurrence (rates between 40% and 75% have been reported after definitive chemoradiation), whereas local relapse is uncommon after trimodal therapy [85, 107, 108]. Sudo *et al* [109] reported that 23% of patients who received bimodal therapy at MD Anderson Cancer Centre had locoregional-only relapse. Therefore, salvage surgery may be considered in a significant proportion of patients.

Long-term survival rate of 17%–51% after salvage oesophagectomy has been reported in several studies [108, 110–112]. In a large European multicentre study, patients submitted to salvage surgery had a 3-year overall survival of 40% and 3-year disease-free survival of 32%, with in-hospital mortality rate of 8% [108].

However, salvage surgery is an option for carefully selected patients, since salvage oesophagectomy is a more morbid operation than either primary oesophagectomy or planned oesophagectomy after neoadjuvant chemoradiotherapy [111]. A pooled analysis of eight studies showed that salvage surgery is associated with a significantly increased incidence of post-operative mortality, anastomotic leak, pulmonary complications and length of hospital stay, when compared to planned oesophagectomy [113].

Surgery should only be attempted if an R0 resection is technically feasible and after distant metastatic disease has been ruled out [108]. More advanced pathologic states (T3–4 or N+), persistent tumour or early relapse after chemoradiotherapy, higher doses of radiation (≥55 Gy) and upper thoracic oesophageal tumours have been associated with worse outcomes after salvage oesophagectomy [110, 111].

### Metastatic disease of oesophageal and OGJ cancer

**What is the best first-line chemotherapy regimen for the treatment of squamous cell carcinoma of the oesophagus?**

***Recommendations***

- First-line treatment with chemotherapy + pembrolizumab is recommended for patients with advanced squamous cell carcinoma and PD-L1 CPS ≥ 10. EVIDENCE LEVEL II, RECOMMENDATION A.
- For patients with lower expression of PD-L1 (CPS < 10), the panel recommends using chemotherapy rather than chemotherapy + immunotherapy, until further data on the role of checkpoint inhibitors in this group are reported. EVIDENCE LEVEL II, RECOMMENDATION C.
- Although cisplatin plus fluorouracil is the most studied regimen to combine with pembrolizumab, the panel believes that the checkpoint inhibitor may also be combined with other fluoropyrimidine and platinum regimens, such as FOLFOX. EVIDENCE LEVEL V, RECOMMENDATION C.
- First-line chemotherapy with a fluoropyrimidine and platinum regimen is suggested if the addition of immunotherapy is not recommended or unavailable. EVIDENCE LEVEL III, RECOMMENDATION A.
- Three-drug cytotoxic regimens such as FLOT or modified DCF should be reserved for young patients with good performance status and high volume of disease. EVIDENCE LEVEL III, RECOMMENDATION B.
- For patients with metastatic squamous cell carcinoma of the oesophagus, incorporation of cetuximab into first-line treatment is not recommended. EVIDENCE LEVEL II, RECOMMENDATION D.

The therapeutic objective in patients with unresectable and/or metastatic oesophageal cancer is to palliate symptoms, including dysphagia, and to improve survival.

Most of the cytotoxic agents were tested in squamous cell carcinoma histology when this histology was predominant. For this reason, chemotherapy regimens for oesophageal cancer employed drugs frequently used in head and neck squamous cell carcinoma, including 5-FU, cisplatin, mitomycin, methotrexate and bleomycin, among others. Many groups adopted the combination of 5-FU and cisplatin as a standard regimen, and since then, studies have looked for a third agent that could add benefit to this regimen.

With the changes in epidemiology, treatments for advanced gastric cancer and oesophageal tumours converged, and thus, most clinical studies from the 1990s on began to include patients with gastric, oesophageal and OGJ cancer independently of the histology. The most important of those studies are described in GTG guidelines for gastric cancer [4].

Phase III KEYNOTE-590 evaluated the role of first-line immunotherapy in oesophageal cancer. The trial enrolled 749 patients with previously untreated advanced oesophageal carcinoma or OGJ Siewert I adenocarcinoma. Patients were randomised to chemotherapy alone (cisplatin + 5-FU for up to six cycles) or in combination with pembrolizumab (200 mg every 3 weeks for up to 35 cycles) [114]. About half the patients had PD-L1 CPS ≥ 10 and 73% of the patients had squamous cell carcinoma. In the interim analysis reported at ESMO 2020, median overall survival (12.4 versus 9.8 months; HR = 0.73; 95% CI = 0.62–0.86; *p* < 0.0001) and median progression-free survival (6.3 versus 5.8 months; HR = 0.65; 95% CI = 0.55–0.76; *p* < 0.0001) were superior in patients treated with pembrolizumab. Confirmed overall response rate was also higher in the experimental arm (45.0% versus 29.3%; *p* < 0.0001). Subgroup analysis of KEYNOTE-590 showed that the benefit of pembrolizumab plus chemotherapy was seen irrespective of the histology (see ‘Which are the first-line regimens recommended for HER2 negative oesophageal or OGJ adenocarcinoma?’ below). Overall survival benefit was seen in the analysis of all included patients, but maximum benefit was observed in patients with CPS ≥ 10. The efficacy of pembrolizumab is patients with CPS < 10 was not reported, so it is possible that high-PD-L1 expressors are inflating the overall survival benefit observed.

**Which are the first-line regimens recommended for HER2 negative oesophageal or OGJ adenocarcinoma?**

**Recommendations**

- For advanced oesophageal or OGJ adenocarcinomas with PD-L1 CPS ≥5, first-line treatment with FOLFOX/CAPOX in association with nivolumab is recommended. EVIDENCE LEVEL II, RECOMMENDATION B.
- Chemotherapy plus pembrolizumab is also an option if PD-L1 CPS ≥ 10. EVIDENCE LEVEL II, RECOMMENDATION C.
- Although cisplatin plus fluorouracil is the most studied regimen to combine with pembrolizumab in oesophagogastric cancer, the panel believes that this checkpoint inhibitor may also be combined to other regimens, such as FOLFOX. EVIDENCE LEVEL V, RECOMMENDATION C.
- For patients with lower expression of PD-L1 (CPS < 5), the panel recommends using chemotherapy rather than chemotherapy + immunotherapy, until further data on the role of checkpoint inhibitors in this group are reported. EVIDENCE LEVEL II, RECOMMENDATION C.
- First-line chemotherapy with a fluoropyrimidine and platinum regimen or FOLFIRI is suggested if the addition of immunotherapy is not recommended or unavailable. EVIDENCE LEVEL I, RECOMMENDATION A.
- Three-drug cytotoxic regimens such as FLOT or modified DCF should be reserved for young patients with good performance status and high volume of disease. EVIDENCE LEVEL II, RECOMMENDATION B.
- First-line pembrolizumab monotherapy is an option for fragile patients or those with low tumour burden, particularly if PD-L1 CPS ≥ 10. EVIDENCE LEVEL II, RECOMMENDATION C.
- Reduced dose CAPOX or single-agent chemotherapy with fluoropyrimidine, irinotecan or paclitaxel are options for patients who are not candidates for aggressive treatment. EVIDENCE LEVEL II, RECOMMENDATION B.
- Immunotherapy alone or in combination with chemotherapy should be the preferential first-line treatment for patients with deficiency in mismatch repair (dMMR)/MSI-H tumours. EVIDENCE LEVEL III, RECOMMENDATION A.

Chemotherapy regimens indicated for metastatic oesophageal, OGJ or gastric adenocarcinoma and metastatic oesophageal squamous cell carcinoma are frequently interchangeably used, although with different levels of evidence. GTG recommendations for the treatment of advanced gastric adenocarcinoma are discussed in detail elsewhere [4].

Briefly, the preferred cytotoxic regimens for the treatment of oesophagogastric adenocarcinomas combine a fluoropyrimidine and a platinum agent. Different phase III trials showed interchangeability between infusional 5-FU and capecitabine and between cisplatin and oxaliplatin [115, 116]. Irinotecan-based regimens such as FOLFIRI and irinotecan + cisplatin are also active as first-line therapy [117–121]. Three-drug cytotoxic regimens, such as DCF, FLOT and TEF, have shown conflicting results in randomised trials and should be reserved for fit patients with high volume of disease and with access to frequent toxicity evaluation [122–125]. A dose-modified DCF regimen improved overall survival with better tolerability in a randomised phase II trial and should be preferred over parental DCF [123]. Most centres abandoned the use of triplets with epirubicin because of the lack of significant benefit over doublets. Low-dose oxaliplatin + capecitabine was shown to be non-inferior to full-dose regimen with better overall treatment utility, a composite of clinical benefit, tolerability, quality of life and patient value, in elderly or frail patients [126]. Therefore, reduced dose fluoropyrimidine + oxaliplatin is a reasonable treatment for patients considered unsuitable for intense chemotherapy. Single-agent chemotherapy with fluoropyrimidine, irinotecan or paclitaxel is also an option for patients who are not candidates for aggressive treatment.

Immunotherapy was incorporated into the treatment of gastroesophageal adenocarcinoma in the past few years and deserves further consideration. The role of nivolumab in the first-line setting was investigated in the phase III trials CheckMate 649 and ATTRACTION-4 [127, 128]. In CheckMate 649, adults with previously untreated HER2-negative advanced oesophageal, OGJ or gastric adenocarcinomas were randomised to receive nivolumab 360 mg every 3 weeks or 240 mg every 2 weeks + chemotherapy (CAPOX or FOLFOX), chemotherapy alone or nivolumab + ipilimumab [127]. Nivolumab + ipilimumab arm was closed and enrolment continued on the other arms. Oesophageal and OGJ cancer patients represented 12% and 18% of the population, respectively. The primary endpoints were overall survival and progression-free survival in patients with PD-L1 CPS ≥ 5. In a preliminary report, nivolumab + chemotherapy showed an improvement in overall survival (14.4 versus 11.1 months; HR = 0.71 (98.4% CI = 0.59–0.86); *p* < 0.0001) and progression-free survival (7.7 versus 6.1 months; HR = 0.68 (98% CI = 0.56–0.81); *p* < 0.0001) when compared to chemotherapy alone in patients with PD-L1 CPS ≥ 5. The addition of nivolumab to chemotherapy also increased response rate (45% versus 60%; *p* < 0.0001). Statistically significant overall survival increase was also observed in patients with PD-L1 CPS ≥ 1 and the all-randomised population. However, the benefit was smaller as PD-L1 expression decreased, so that the impact of nivolumab in patients with CPS 1–5 and, especially, in patients with PD-L1 0 is uncertain. Further analysis of biomarker selected patients is required to understand the value of nivolumab for all patients. In overall survival subgroup analysis, the impact of nivolumab was somewhat less impressive in oesophageal or OGJ adenocarcinoma (HR = 0.78 and 0.84, respectively) when compared to gastric cancer (HR = 0.66) [127].

ATTRACTION-4 randomised 724 Asian patients with gastric or OGJ adenocarcinoma to receive nivolumab plus chemotherapy (S-1 + oxaliplatin or capecitabine + oxaliplatin) or placebo + chemotherapy [128]. The primary endpoints were centrally assessed progression-free survival and overall survival. At the interim analysis, median progression-free survival increased from 8.3 to 10.5 months in the experimental arm (HR = 0.68; 98.51% CI = 0.51–0.90; *p* = 0.0007), but no overall survival difference was found (HR = 0.90; 95% CI = 0.75–1.08; *p* = 0.257). Increased use of second-line treatment and of post-progression checkpoint inhibitors in ATTRACTION-4 patients, as compared to CheckMate 649, may help to justify the lack of overall survival benefit. PD-L1 expression evaluated by TPS did not predict benefit of nivolumab in ATTRACTION-4, in contrast to CPS used in CheckMate 649 or KEYNOTE-590 trials [114, 127, 128]. The role of pembrolizumab, another anti-PD-1 monoclonal antibody, in gastroesophageal carcinoma was also evaluated in phase III trials. KEYNOTE-062 randomised 763 patients with untreated advanced gastric or OGJ adenocarcinoma with PD-L1 CPS ≥ 1 to pembrolizumab 200 mg, pembrolizumab + chemotherapy (cisplatin + fluorouracil or capecitabine) or chemotherapy + placebo every 3 weeks [129]. OGJ adenocarcinoma represented about 30% of included patients. Primary endpoints were overall survival and progression-free survival in patients with PD-L1 CPS ≥ 1 or ≥ 10. In contrast to CheckMate 649, immunotherapy plus chemotherapy was not superior to chemotherapy plus placebo, irrespective of the magnitude of PD-L1 expression. On the other hand, interesting results were found in pembrolizumab monotherapy arm. The authors reported that pembrolizumab was non-inferior to chemotherapy for overall survival in patients with CPS ≥1 (median 10.6 versus 11.1 months; HR = 0.91; 99.2% CI = 0.69–1.18), with better tolerability. In an exploratory analysis of patients with CPS ≥10, overall survival was numerically superior in pembrolizumab arm (median 17.4 versus 10.8 months; HR = 0.69; 95% CI = 0.49–0.97), but this difference was not statistically tested [129]. First-line pembrolizumab monotherapy may be an option for carefully selected patients with PD-L1 expression. However, since short progression-free survival and low response rate were observed with pembrolizumab alone, that strategy may be restricted for fragile patients or those with low tumour burden, particularly if PD-L1 CPS ≥ 10.

The KEYNOTE-590 trial observed an increase in overall survival in patients with oesophageal carcinoma treated with first-line chemotherapy plus pembrolizumab when compared to chemotherapy alone, particularly if CPS ≥ 10 [114] (see ‘What is the best first-line chemotherapy regimen for the treatment of squamous cell carcinoma of the oesophagus?’ above). Subgroup analysis of KEYNOTE-590 showed that the benefit of pembrolizumab plus chemotherapy was seen irrespective of the histology. However, only 27% of the patients included in KEYNOTE-590 were adenocarcinomas, and previously discussed KEYNOTE-062 trial did not show impact of the addition of pembrolizumab to first-line chemotherapy in advanced gastroesophageal adenocarcinoma [129]. Therefore, the positive results of KEYNOTE-590 adenocarcinoma subgroup may be interpreted cautiously.

dMMR/MSI-H patients are overly sensitive to immunotherapy. Subset analyses of CheckMate 649 and KEYNOTE-062 trials suggest that the use of a checkpoint inhibitor in the treatment of MSI-H patients provides survival benefit over control [127, 129]. In previously described KEYNOTE-062, the survival benefit was enhanced with pembrolizumab (HR 0.29) and pembrolizumab plus chemotherapy (HR 0.37) versus chemotherapy in MSI-H patients [129]. The panel believes that immunotherapy alone or in combination with chemotherapy should be the preferential treatment for dMMR/MSI-H patients.

**Which are the first-line regimens recommended for HER2 positive oesophageal, OGJ or advanced gastric adenocarcinoma?**

***Recommendation***

- For patients with OGJ metastatic adenocarcinoma who have HER2 overexpression (3+ in IHC or FISH + or 2+ in IHC and confirmed by FISH+), it is recommended to use trastuzumab in the first-line chemotherapy regimen with a combination of platinum and fluoropyrimidine. EVIDENCE LEVEL I, RECOMMENDATION A.

The benefit of trastuzumab, an anti-HER2 monoclonal antibody, in adenocarcinomas of the stomach and OGJ, was evidenced in a phase III study called ToGA, which compared cisplatin and 5-FU/capecitabine regimens with and without trastuzumab (8 mg/kg in the first cycle and 6 mg/kg in the remaining cycles). Objective response rates were significantly higher in the trastuzumab-treated group (47% versus 35%). After a median follow-up of 1.5-year, the median overall survival was also significantly superior in the trastuzumab-treated group (13.8 versus 11.1 months) [130].

Based on the TOGA trial, trastuzumab should be incorporated into the therapy of patients with gastric and OGJ adenocarcinoma who have overexpression of HER2. Considering that the biology background of metastatic oesophageal adenocarcinoma is very similar to metastatic adenocarcinomas of the OGJ and stomach, the use of trastuzumab should be extended to patients with these tumours.

Other anti-HER2 agents, such as pertuzumab and lapatinib, are not effective in the treatment of gastroesophageal cancer [131, 132]. Similarly, maintenance of trastuzumab beyond first-line progression and higher than standard doses of trastuzumab are also not recommended [131, 133].

A single-arm study with 37 patients evaluated the activity of the addition of pembrolizumab to trastuzumab + chemotherapy in first-line treatment of HER2-positive metastatic oesophagogastric cancer patients [134]. The median progression-free survival was 13.0 months, median overall survival was 27.3 months and 91% of patients with measurable disease achieved overall response. Phase III trial of this combination compared to standard of care, KEYNOTE-811, is ongoing.

What are the recommended regimens for the treatment of second and subsequent lines for oesophageal squamous cell carcinoma?

***Recommendations***

- Patients with PD-L1 CPS ≥10 squamous cell carcinoma may be treated with second-line pembrolizumab. EVIDENCE LEVEL I, RECOMMENDATION A
- Nivolumab may be used as second-line treatment of advanced squamous cell oesophageal cancer, regardless of biomarker expression. EVIDENCE LEVEL II, RECOMMENDATION B.
- Patients may receive second-line monotherapy with irinotecan or taxane for metastatic squamous cell carcinoma of the oesophagus. EVIDENCE LEVEL III, RECOMMENDATION B.
- Patients with preserved *performance status* and previously treated with two lines may receive subsequent treatment with active drugs not yet used. EVIDENCE LEVEL V, RECOMMENDATION C.
- Pembrolizumab may be indicated for second-line or subsequent therapy for MSI-H tumours. EVIDENCE LEVEL III, RECOMMENDATION A
- Larotrectinib should be considered in *NTRK*-gene fusion-positive patients. EVIDENCE LEVEL III, RECOMMENDATION A

There are only limited data on the efficacy of second-line chemotherapy for advanced oesophageal cancer. Therefore, there is no standard approach, and the choice of therapy regimen is eventually empirical.

The phase III KEYNOTE-181 study compared pembrolizumab (200 mg every 3 weeks) or the investigators’ choice of chemotherapy (docetaxel, paclitaxel or irinotecan) as second-line therapy for patients with advanced squamous cell carcinoma or adenocarcinoma of the oesophagus or OGJ (Siewert type I) [135]. The primary outcome was overall survival in patients with squamous cell carcinoma, patients with tumours expressing PD-L1 CPS ≥ 10, and all randomised patients. Among the 222 patients who had PD-L1 CPS ≥ 10, pembrolizumab significantly improved median overall survival (9.3 versus 6.7 months) and overall response rate (22% versus 6%). There was also significant improvement in overall survival with pembrolizumab in squamous cell carcinoma subgroup (8.2 versus 7.1 months). Fewer patients had grade 3–5 treatment-related adverse events with pembrolizumab compared to chemotherapy.

The role of nivolumab as second-line treatment of advanced squamous cell oesophageal carcinoma was evaluated in ATTRACTION-3 trial [136]. In this study, 419 patients who were refractory or intolerant to one previous fluoropyrimidine-based and platinum-based chemotherapy were randomised to receive either nivolumab (240 mg every 2 weeks) or investigator’s choice of chemotherapy (paclitaxel 100 mg/m^2^ per week for 6 weeks then 1 week off; or docetaxel 75 mg/m^2^ every 3 weeks). 96% of enrolled patients were Asian. Overall survival was significantly improved in the nivolumab group (median 10.9 months versus 8.4 months; HR = 0.77; 95% CI = 0.62–0.96; *p* = 0.019). The benefit was seen irrespective of tumour PD-L1 expression (TPS), as evaluated by Dako 28-8 pharmDx assay [136].

Le *et al* [77] evaluated the activity of pembrolizumab in a cohort of 78 patients with advanced mismatch repair-deficient cancers across 12 different tumour types, from which five had gastroesophageal primaries. Objective responses were observed in 53% of patients. Responses were durable, with median progression-free survival and overall survival still not reached. The activity of pembrolizumab in MSI-H tumours gave the drug the first approval of a tissue-site agnostic agent by Food and Drug Administration (FDA) [137].

Patients with *NTRK* fusion-positive tumours of different primary sites had a response rate of 75% after treatment with larotrectinib. The progression-free survival rate at 1 year was 55%. Although exceptionally rare in oesophagogastric tumours, NTRK-fusion testing is recommended to identify eventual candidates to NTRK inhibitors [138].

**Which regimens are recommended for the treatment of second and subsequent lines of oesophageal or OGJ adenocarcinoma?**

***Recommendations***

- In the metastatic setting, patients with oesophageal or OGJ adenocarcinoma are usually treated similarly to patients with gastric adenocarcinoma.
- Second-line chemotherapy should be recommended for patients with good performance status (ECOG 0–1). EVIDENCE LEVEL I, RECOMMENDATION A.
- Paclitaxel associated with ramucirumab, when available, is recommended for patients with ECOG 0–1. EVIDENCE LEVEL II, RECOMMENDATION A.
- Patients with oesophageal adenocarcinoma and PD-L1 CPS ≥10 may be treated with second-line pembrolizumab. EVIDENCE LEVEL II, RECOMMENDATION B.
- Monotherapy with ramucirumab, irinotecan or taxane are alternatives as second or later treatment of advanced oesophagogastric adenocarcinoma. EVIDENCE LEVEL II, RECOMMENDATION B.
- Third or later lines of chemotherapy may be used in patients with metastatic oesophagogastric adenocarcinoma, depending on previous regimens and performance status. EVIDENCE LEVEL IV, RECOMMENDATION C.
- Pembrolizumab may be used in the third-line or subsequent treatments of patients with ECOG 0 or 1 and PD-L1-positive gastric or OGJ adenocarcinoma. EVIDENCE LEVEL III, RECOMMENDATION A.
- Pembrolizumab is indicated for second or subsequent lines of treatment in patients with advanced gastric or OGJ adenocarcinoma and MSI not previously treated with checkpoint inhibitors, although this indication has not yet been approved in Brazil. EVIDENCE LEVEL III, RECOMMENDATION A.
- Larotrectinib should be considered in *NTRK*-gene fusion-positive patients. EVIDENCE LEVEL III, RECOMMENDATION A.
- Trastuzumab deruxtecan may be considered in trastuzumab-resistant HER2-positive OGJ adenocarcinoma, if available. EVIDENCE LEVEL II, RECOMMENDATION A.

See GTG gastric cancer consensus for discussion about chemotherapy, NTRK inhibitors, trastuzumab deruxtecan and immunotherapy in PD-L1 CPS ≥ 1 or MSI-H patients [4]. The aforementioned KEYNOTE-181 showed that second-line pembrolizumab increased overall survival over chemotherapy in patients with oesophageal carcinomas and PD-L1 CPS ≥ 10. However, subgroup analysis identified that the benefit was mostly driven by squamous cell carcinoma patients. Second-line pembrolizumab in patients with adenocarcinoma and PD-L1 CPS ≥ 10 is approved by Brazilian Health Regulatory Agency (ANVISA), but not by FDA [135].

### Oesophageal and OGJ cancer follow-up

**How should patients with oesophageal, OGJ and gastric cancer be followed-up after treatment with curative intent?**

For patients with cancer of the oesophagus or OGJ, T1s or T1a treated with endoscopic resection or ablation: [139] UDE every 3 months in the first year and every 6 months in the second year. After this period, annual UDE for 5 years. EVIDENCE LEVEL IV, RECOMMENDATION B.

- Imaging exams are not recommended. EVIDENCE LEVEL V, RECOMMENDATION A.
- For patients with oesophageal or OGJ cancer, T1s or T1a treated with oesophagectomy:
- UDE should be carried out according to symptoms. EVIDENCE LEVEL V, RECOMMENDATION A.
- Imaging exams are not recommended. EVIDENCE LEVEL V, RECOMMENDATION A.

For patients with oesophageal or OGJ cancer, T1bN0 treated with chemoradiotherapy:

- UDE should be carried out every 6 months for the first 3 years. Repeat UDE only if clinically indicated. EVIDENCE LEVEL IV, RECOMMENDATION A;
- CT of the chest and abdomen can be considered every 6–12 months in the first three years, then if clinically indicated. EVIDENCE LEVEL V RECOMMENDATION C.

For patients with oesophageal or JEG cancer, T2–4 or N+ treated with chemoradiotherapy:

- UDE may be carried out every 6 months for the first 3 years, then if clinically indicated. EVIDENCE LEVEL IV RECOMMENDATION A.
- CTs of the chest and abdomen can be considered every 6–12 months for the first 2 years. After this period, CT can be carried out annually until the fifth year. EVIDENCE LEVEL V RECOMMENDATION C.

For patients with oesophageal or OGJ cancer, T2–4 or N+ treated with trimodal therapy:

- UDE may be carried out, if clinically indicated. EVIDENCE LEVEL V RECOMMENDATION A.
- CT of the chest and abdomen can be considered every 6–12 months for the first 2 years. After this period, CT may be carried out annually until the fifth year. EVIDENCE LEVEL V RECOMMENDATION C.

## Conclusion

Scientific evidence must endorse decisions in daily medical practice. Cancer patients present complex requirements that are often difficult to be addressed by a single specialist. The GTG suggests a multidisciplinary approach and monitoring of patients with oesophageal and OGJ cancer. In line with this, GTG developed local specialist consensus guidelines on oesophageal and OGJ cancer. Nonetheless, the therapeutic choice of the physician must always prevail, considering the individuality of the patient.

## Funding source

There were no funding sources for this project.

## Conflicts of interest

Duilio R Rocha-Filho: personal fees from Bayer, Merck Serono and Servier.

Renata D’Alpino Peixoto: personal fees from Lilly and MSD.

Anelisa K Coutinho: personal fees from Amgen, Bayer, Roche, MSD, Merck Serono, Sanofi, Servier, Ipsen, Novartis and United Medical.

Juliana F M Rego: personal fees from Novartis, Bayer, Amgen and Ipsen.

Rachel Riechelmann: research grants from Libbs (gastric consensus only).

Rui F Weschenfelder: personal fees from Lilly, MSD and Roche.

Alexandre A Jacome: personal fees from Roche, Novartis, Zodiac and Pfizer. Research grants from Bayer.

Celso A L Mello: personal fees from Lilly, Merck Serono, MSD and Roche.

Gustavo S Fernandes: personal fees from BMS, Bayer and Roche.

Roberto A Gil: personal fees from Amgen, Bayer, Lilly, Merck Serono, Roche, Sanofi and United Medical. Research grant from Amgen, Bayer, Merck and Roche.

Victor Hugo F Jesus: personal fees from United Medical.

## Figures and Tables

**Table 1.** Level of evidence and strength of recommendation. CDC classification system.

I	Evidence of at least one large randomised controlled trial (RCT), of good methodological quality (low potential for bias) or meta-analyses of well-conducted randomised clinical trials without heterogeneity
II	RCTs^a^ small or large RCTs^a^ with suspicion of bias (low methodological quality) or meta-analyses of these tests or tests with demonstrated heterogeneity
III	Prospective cohort studies
IV	Retrospective cohort studies or case–control studies
V	Studies without a control group, case reports and expert opinions

A	Solid evidence of efficacy with substantial clinical advantage – strongly recommended
B	Strong or moderate evidence, in terms of effectiveness, but with a limited clinical benefit – generally recommended
C	Insufficient evidence of effectiveness or benefit does not outweigh risks/disadvantages (i.e. adverse events or costs) – optional
D	Moderate evidence against effectiveness or evidence indicating adverse outcomes – generally not recommended
E	Strong evidence against effectiveness or indicating adverse outcomes – not recommended

a RCTs, Randomised clinical trials

**Table 2.** Ryan’s modified system for therapeutic response.

Description	Score
No viable cancer cells (complete response)	0
Isolated cells or rare small groups of cancer cells (almost complete response)	1
Residual cancer with evident tumour regression, however, with more than single cells or rare small groups of cancer cells (partial response)	2
Extensive residual cancer without evident tumour regression (low or no response)	3

**Table 3.** Score system for HER2 assessment in gastric and gastroesophageal adenocarcinoma.

Score	Microscope magnification	Staining pattern of surgical specimen	Staining pattern of biopsy	HER2 overexpression assessment
0	40×	No reactivity or membranous reactivity in <10% of tumour cells	Absence of reactivity or absence of membranous reactivity	Negative
1+	40×	Subtle or barely detectable membranous reactivity in ≥10% of tumour cells. Reactivity only in part of the cell membrane	Group of tumour cells with subtle or barely notable membrane reactivity, regardless of the percentage of reactive cells	Negative
2+	10–20×	Weak to moderate membranous reactivity, complete, basal lateral or lateral in ≥10% of tumour cells	Group of tumour cells with weak to moderate complete basolateral or lateral membrane reactivity, regardless of the percentage of reactive cells	Undetermined (need confirmation with FISH or SISH)
3+	2.5–5×	Strong and complete membranous reactivity, basolateral or lateral in ≥10% of tumour cells	Group of tumour cells with membranous reactivity, basolateral or lateral independently of the reactive cells’ percentage	Positive

SISH: silver-enhanced in situ hybridization; HER: human epidermal growth factor receptor; FISH: fluorescence in situ hybridization
